# A Cross-Sectional Study of the Impact of COVID-19 on Domestic Violence, Menstruation, Genital Tract Health, and Contraception Use among Women in Jordan

**DOI:** 10.4269/ajtmh.20-1269

**Published:** 2020-12-29

**Authors:** Iman Aolymat

**Affiliations:** Department of Basic Medical Sciences, Faculty of Medicine, The Hashemite University, Zarqa, Jordan

## Abstract

The COVID-19 pandemic is a major global concern for public health where high numbers of COVID-19–infected cases and deaths have been recorded. This study assessed the COVID-19 pandemic impact on domestic violence, genital tract health, menstruation, and contraception use among 200 women in Jordan using a self-validated survey questionnaire. The questionnaire was structured to compare frequencies of domestic violence, reproductive tract infections, menstrual irregularities, and contraception use, type, source, and replacement during or after total curfew in Jordan with 6-months before the pandemic; 20.5% of women suffered from increased domestic abuse during the COVID-19 pandemic. Incidence of menstrual problems and genital tract infections was significantly reduced during total curfew compared with 6 months prior (10.5% versus 17.5%; *P* = 0.016 and 19% versus 25.5%; *P* = 0.041, respectively). Pre-pandemic state of menstrual problems and genital tract infections was resumed after total curfew. During total curfew, phone consultations were significantly increased (17.5% versus 8.5%; *P* = 0.01), whereas visiting clinics was significantly reduced (23% versus 5.5%; *P* = 0.000) to manage menstruation or birth canal infections. Contraception use during total curfew significantly decreased compared with prior (59.5% versus 65.5%; *P* = 0.017). Using contraception for family planning was reduced significantly during the pandemic than before (*P* = 0.007). Maternity and childhood centers were more common sources for contraception before than after (14.8% versus 7% or 9.5%; *P =* 0.001 or *P =* 0.022). This study is important to evaluate preparedness of Jordanian healthcare systems in facing pandemic situations concerning reproductive health services.

## INTRODUCTION

The most recently discovered member of the coronaviruses family called SARS-CoV-2 is associated with an emerging respiratory infection.^1^ Coronaviruses are known infectious pathogens in humans and animals. In humans, the disease manifests as a respiratory tract infection extending from the common cold to more serious respiratory syndromes.^2^ Middle East respiratory syndrome coronavirus (MERS-CoV) and SARS-CoV are previously identified viruses similar to novel SARS-CoV-2.^2^ COVID-19 was originally discovered in Wuhan, China, in September 2020, and it was declared as a Public Health Emergency of International Concern on January 30, 2020 and a global pandemic by the WHO on March 11, 2020.^2^ As of the time of writing, SARS‐CoV‐2 had infected more than 78 million people and caused more than 1.7 million deaths.^2^ The United States has the world’s highest number of COVID-19–infected cases and deaths.^3^ Jordan has confirmed more than 282 thousand infected cases and 3652 COVID-19–related deaths since the beginning of the crisis.^2^ COVID-19 is transmitted from person to person directly through contact with emitted viral particles from the respiratory tract of an infected individual or indirectly through virus-contaminated surfaces.^2^ Clinically, COVID-19 presents with a range of symptoms extending from asymptomatic, to fever, dry cough, and tiredness to more serious or lethal diseases with shortness of breath and severe acute respiratory syndrome.^1^ Up to date, no available curable treatment or vaccine for the diseases is available. Substantial research efforts have been conducted to develop a curative treatment or vaccine for this disease; however, it was not successful, unfortunately, and infected patients are treated symptomatically only.

The WHO recommended that sexual and reproductive health services, including family planning, are fundamental services to be continued during the COVID-19 outbreak.^4^ The COVID-19 pandemic is already stretching the healthcare systems around the world by putting more demands for caring for COVID-19–infected patients. On the other hand, the other routine reproductive health services such as family planning were stopped completely or limited during the pandemic because of the shortness of healthcare providers who have been converted to treat COVID-19 patients or because of lockdown procedures to contain the COVID-19 pandemic. The defect in family planning and other reproductive health services is expected to be associated with a greater risk of health problems and deaths. Many previous epidemiologic researches tackled the relationship between chronic stress and disruption in physiological process required for fertility (such as menstrual cycle), genital tract health, and contraception use.^5–10^ To the best of my knowledge, no previous studies specifically investigated the impact of the COVID‐19 pandemic as a stressful event on family planning and reproductive physiology in Jordan. Therefore, the present study aimed to examine the influence of the COVID‐19 pandemic on domestic violence, menstruation, genital tract health, and contraception use among women in Jordan. This study provides an opportunity to evaluate the extent of the readiness of Jordanian healthcare systems to face pandemic situations with regard to reproductive health services.

## MATERIALS AND METHODS

### Sample and data collection.

In this study, a cross-sectional study was conducted in Jordan between September 1 and 8, 2020. The sample consisted of 200 women. The data were collected using an online self-administered survey. The questionnaire was originally written in English, and then translated to Arabic with the help of a professional Arabic translator. The content of the questionnaire was validated by conducting pilot testing on 30 participants, who were asked to comment on the items of the questionnaire in terms of clarity and reliability. The pilot test was excluded from the final analysis. After the pilot test was finished, further revisions and amendments to make the survey clear and reliable were conducted based on the pilot test participants’ feedback. Consequently, the online survey was created using Google Forms, and invitation for married women aged 18 years or older to complete a 10-minute online survey was posted on social media platforms. The questionnaire included an introduction showing the goals of the study and emphasizing on the incompulsory participation in this survey, anonymity, and confidentiality of the participants. The questionnaire composed of six sections, including short-answer or multiple-choice questions. The first section included the demographic characteristics of the study participants such as age, education level, place of residence, age at first menstruation, and marriage, gravidity, and some obstetric information. The second part involved questions about weight, menstruation, and reproductive canal health problems during the COVID-19 pandemic. The third section consisted of questions about using, types, purposes, and sources of contraception during the COVID-19 pandemic and relevant problems to contraception use during the COVID-19 pandemic. The remaining sections including questions about pregnancy and childbirth, sexual behavior, and reproductive attitudes have been submitted for publication elsewhere.

### Ethics approval.

The current study has been reviewed and approved by the Institutional Review Board Committee of the Hashemite University (August 25, 2020). In addition, an informed consent form on the first page of the online questionnaire indicating the optional participation in this study was obtained before progression to questionnaire sections.

### Statistical analysis.

Data retrieved from the online survey were imported into Microsoft Excel (Microsoft Corporation, Redmond, WA) and then into the Statistical Package for Social Sciences (SPSS) version 25 (IBM Corporation, Armonk, NY) for analysis. Output measures were portrayed as simple frequency (*n*) and percentage (%) or as means and SDs. The McNemar test and paired sample *t*-test were used to compare the percentages and averages. Statistically significant differences were considered when *P* < 0.05.

## RESULTS

### Demographic characteristics of participants.

Two hundred married women 18 years or older participated in this study. The majority of the women (70%) were within the ages of 25–34 years. More than two-thirds of the participants (70%) were holders of undergraduate degrees. The remaining 25.5% and 4.5% of women had enrolled in postgraduate and secondary school or less education, respectively; 154 participants live in urban places, whereas 46 participants live in rural areas. The mean age at first menstruation was 13.48 years, and the mean age at marriage was 24 years. Other demographic characteristics such as gravidity, number of abortions, vaginal births, cesarean deliveries, and proportion of participants with chronic diseases are shown in Table 1.

**Table 1:** Demographic characteristics of the study participants (*n* = 200)

Demographic variable	Mean ± SD or % (*n*)
Age-group (years)	
18–24	2.5 (5)
25–34	70.0 (140)
35–44	19.5 (39)
≥ 45	8.0 (16)
Education level	
Secondary or less	4.5 (9)
Undergraduate	70.0 (140)
Postgraduate	25.5 (51)
Place of residence	
Urban	77.0 (154)
Rural	23.0 (46)
Age at first menstruation (years)	13.48 ± 1.8
Age at marriage (years)	24.0 ± 3.1
Gravidity	2.9 ± 1.9
No. of abortions	0.5 ± 0.8
No. of vaginal births	1.7 ± 1.5
No. of cesarean deliveries	0.7 ± 1.0
Chronic diseases	
No	93.5 (187)
Yes	6.5 (13)

### Impact of COVID-19 on domestic violence, weight, reproductive tract infection, and menstruation.

In total, 20.5% of the participants suffered from increased domestic abuse during the COVID-19 pandemic. Average weight of the participants was significantly increased during the total curfew and after the total curfew compared with the 6 months before COVID-19 (70.1% and 70% versus 68.6%; *P* = 0.000). Before the pandemic, 17.5% of the women had menstrual aberrations; however, during the total curfew, this proportion was decreased to 10.5% (*P* = 0.016). After the total curfew, the proportion of the participants with menstrual disorders was not statistically different from that before the pandemic (13% versus 17.5%, *P =* 0.163). Furthermore, before the beginning of the COVID-19 pandemic, 51 women had gynecological infections; however, during the total curfew, 38 participants complained from symptoms of gynecological infections. These values were statistically distinguishable, indicating that the total curfew was associated with decreased reproductive tract infections. Nevertheless, after the total curfew, the number of women with reproductive tract infection was not statistically different from that observed in the pre-pandemic state (Table 2).

**Table 2:** Comparison of participant’s responses about the effect of COVID-19 on weight, menstruation, and reproductive canal health

	Six months before COVID-19 (control)	During total curfew	*P*-value	After total curfew	*P*-value
Weight	68.6 ± 11.8	70.1 ± 12.1	0.000	70.0 ± 11.7	0.000
Menstrual disorders, % (*n*)	17.5 (35)	10.5 (21)	0.016	13.0 (26)	0.163
Genital tract infection, % (*n*)	25.5 (51)	19.0 (38)	0.041	20 (40)	0.126

By analyzing the ways used to treat menstrual disorders and gynecological infections, the proportions of menstrual disorders or genital tract infections that had never been treated before the pandemic (21%), during the total curfew (29%), and after the pandemic (24.5%) were not different. Conversely, using phone consultation to obtain a proper medical care was significantly doubled during the total curfew duration (8.5% versus 17.5%; *P* = 0.01). However, using phone consultation to support remote management of participants after the total curfew was not different from the pre-pandemic state (8% versus 8.5; *P* = 0.865)*.* Visiting the clinics during the total curfew was dramatically reduced by four times in comparison with the visits conducted before the pandemic (5.5% versus 23%; *P* = 0.000). After the total curfew, the pre-pandemic clinic visiting state was resumed to treat menstrual irregularities or reproductive tract infection (22% versus 23%; *P* = 1.00). Receiving medical care through the pharmacies during or after the total curfew (10%) was similar to that of the pre-pandemic era (12.5%). Eleven women used other management options (such as herbal treatments and traditional medicine) before the start of the COVID-19 pandemic, whereas 13 and 11 women used this option to treat menstrual disruption or reproductive tract infections during and after the total curfew, respectively (Table 3).

**Table 3:** Comparison of participant’s responses about management of menstrual disorders/gynecological infection before and during the COVID-19 pandemic

	Six months before COVID-19 (control), % (*n*)	During total curfew, % (*n*)	*P*-value	After total curfew, % (*n*)	*P*-value
Never been treated	21.0 (42)	29.0 (58)	0.068	24.5 (49)	0.464
Phone consultation	8.5 (17)	17.5 (35)	0.01	8.0 (16)	0.865
Going personally to pharmacy for treatment	12.5 (25)	10.0 (20)	0.5	10.0 (20)	0.542
Going personally to clinics for treatment	23.0 (46)	5.5 (11)	0.000	22.0 (44)	1.00
Others	5.5 (11)	6.5 (13)	0.832	5.5 (11)	1.00

### Impact of COVID-19 on contraception use.

Table 4 represents the change in using contraception during the COVID-19 pandemic. Before COVID-19 pandemic appearance in Jordan, 65.5% of the participants used contraception, but the proportion of the respondents using contraception during the total curfew was significantly reduced to 59.5% (*P* = 0.017). After the total curfew, 61.5% reported using contraception, which was similar to what was observed before the pandemic. Family planning was the major aim for using contraception before and after the pandemic. The proportions of the respondents using contraception for family planning before COVID-19, during total curfew, and after the total curfew were as follows: 55%, 48.5%, and 47.5%, respectively. The proportion of respondents using contraception for family planning purpose during and after the total curfew was significantly reduced (*P =* 0.007). Therapeutic use of contraception was the least common in this study because only 0–0.5% of the participants used contraception to treat certain disorders before and after the COVID-19 pandemic; 3–3.5% of the women participated in this study used contraception before and after the COVID-19 pandemic to prevent pregnancy-related health risks for women. To protect against sexually transmitted disease (STDs), the numbers of women were as follows: two of them used contraception to protect against STDs before COVID-19 pandemic, only one woman used contraception to protect against STDs during the total curfew, and three participants applied contraception to reduce the risk of STDs after the total curfew. Interestingly, four and five women used contraception during and after total curfew because of fears from possible COVID-19–related negative impacts on pregnant or fetus health; 12 women used contraception before, 10 women used contraception during total curfew, and 13 women used contraception after total curfew for other reasons such as to prevent cycles during sports, parties, and fasting and other worships.

**Table 4:** Comparison of participant’s responses about use, purposes, and types of contraception before and after COVID-19 pandemic

	Six months before COVID-19 (control), % (*n*)	During total curfew, % (*n*)	*P*-value	After total curfew, % (*n*)	*P*-value
Using contraception	65.5 (131)	59.5 (119)	0.017	61.5 (123)	0.215
Reason for contraception use					
Family planning	55.0 (110)	48.5 (97)	0.007	47.5 (95)	0.007
Therapeutic	0.5 (1)	0.0 (0)	NA	0.5 (1)	NA
High-risk pregnancy	3.0 (6)	3.0 (6)	1.00	3.5 (7)	1.00
Protection against sexually transmitted diseases	1.0 (2)	0.5 (1)	NA	1.5 (3)	NA
Fear from COVID-19 negative impact on mother/fetus	0.0 (0)	2.5 (5)	NA	2.0 (4)	NA
Others	6.0 (12)	5.0 (10)	0.5	6.5 (13)	0.5
Type of contraception					
Contraceptive pills	12.5 (25)	10.5 (21)	0.219	9.0 (18)	0.065
Implants	0.0 (0)	0.0 (0)	NA	0.0 (0)	NA
Injection	0.0 (0)	0.0 (0)	NA	0.5 (1)	NA
Intra-uterine device	18.0 (36)	16.0 (32)	0.125	16.5 (33)	0.435
Male condoms	15.5 (31)	15.0 (30)	1.000	15.0 (30)	1.000
Female condoms	0.0 (0)	0.0 (0)	NA	0.0 (0)	NA
Fertility awareness	5.5 (11)	6.0 (12)	1.000	7.0 (14)	0.508
Breastfeeding	0.0 (0)	0.0 (0)	NA	0.0 (0)	NA
External ejaculation	14.0 (28)	12.0 (24)	0.289	13.0 (26)	0.774
Female sterilization	0.0 (0)	0.0 (0)	NA	0.5 (1)	NA

NA = not applicable.

Table 4 also represents the type of contraception used by women before and after the beginning of the COVID-19 pandemic. The most common contraception type used before and after the COVID-19 pandemic in this study was intra-uterine device (IUD) followed by male condoms. On the other hand, none of the women involved in the study used implants or breastfeeding as contraception techniques. By analyzing the types of contraception used by the women in the three periods of the study (before pandemic, during, and after the total curfew), it was observed that there was no significant change in the proportion of women using particular contraception technique before and after the pandemic. Of the 119 women using contraception during the total curfew, 24 women changed their contraception method. Their satisfaction about changing contraception was as follows: one woman was extremely dissatisfied, four women were dissatisfied, nine participants were neither dissatisfied nor satisfied, nine women were satisfied, and one woman was extremely satisfied (Table 5). After the total curfew, 28 participants of 123 women using contraception changed their contraception type. Two participants were extremely dissatisfied, two women were dissatisfied, 11 of them were neither dissatisfied nor satisfied, eight women were satisfied, and five women were extremely satisfied.

**Table 5:** Proportion of participants who changed their contraception during the COVID-19 pandemic and their satisfaction with that change

	During total curfew, % (*n*)	After total curfew, % (*n*)
No contraception	40.5 (81)	38.5 (77)
Extremely dissatisfied	0.5 (1)	1.0 (2)
Dissatisfied	2.0 (4)	1.0 (2)
Neither dissatisfied nor satisfied	4.5 (9)	5.5 (11)
Satisfied	4.5 (9)	4.0 (8)
Extremely satisfied	0.5 (1)	2.5 (5)
Contraception did not change	47.5 (95)	47.5 (95)

Table 6 shows comparison of the participants’ proportions using different sources of contraception in Jordan before and after the COVID-19 pandemic. Most of the women using contraception either did not need external sources for contraception (19%) or obtained their contraception from the pharmacies (18.5%) before the COVID-19 pandemic. About 15% of the women also obtained their pre-pandemic contraception from maternity and childhood centers or health centers which are governmental resources that offer contraception for nominal costs. Interestingly, the proportion of women using the governmental resources was significantly reduced to 7% (*P =* 0.001) during the total curfew and to 9.5% (*P =* 0.022) after the total curfew. The proportion of women using pharmacies and private clinics to obtain their contraception did not change during the pandemic if compared with the pre-pandemic state. It was observed that the proportion of women storing extra contraception was significantly increased during the total curfew in comparison to the era of pre–COVID-19 (5.5% versus 1.5%; *P* = 0.021). The proportion of women who had extra contraception after the total curfew was not statistically distinguishable from that before (2.5% versus 1.5%). Among the 200 participants, two women reported that they were not able to find a source for their contraception because of total curfew. Of the 200 women located, none of the women reported that there was a lack of medical care for contraception-related complications before the pandemic; however, 11 women were unable to obtain a medical care to manage their complications linked with contraception use during the total curfew, and eight women were unable to obtain reproductive health services to treat contraception-related complications after the total curfew.

**Table 6:** Comparison of participant’s responses about source of contraception and availability of medical care for contraception-related complications before and after the COVID-19 pandemic

	Six months before COVID-19 (control), % (*n*)	During total curfew, % (*n*)	*P*-value	After total curfew, % (*n*)	*P*-value
Source of contraception					
Maternity and childhood centers/health centers	14.8 (28)	7.0 (14)	0.001	9.5 (19)	0.022
Pharmacies	18.5 (37)	16.5 (33)	0.481	17.0 (34)	0.607
Private clinics	9.5 (19)	8.5 (17)	0.500	9.0 (18)	1.000
Method used does not need external source	19.0 (38)	17.5 (35)	0.549	19.5 (39)	1.000
No need to renew contraception (e.g., it is long acting)	3.0 (6)	3.5 (7)	1.000	4.0 (8)	0.625
Have extra contraception	1.5 (3)	5.5 (11)	0.021	2.5 (5)	0.687
No available source because of total lockdown	0.0 (0)	1.0 (2)	NA	0.0 (0)	NA
No medical care for contraception complications	0.0 (0)	5.5 (11)	NA	4.0 (8)	NA

NA = not applicable.

During the total curfew, eight of the participants needed to replace their IUD and seven of them were not able to renew their IUD. After the total curfew, five women were in need to renew or replace their contraceptive injections or IUD, but two of them were unable to do that. Nevertheless, the proportion of women who were unable to change their IUD or repeat their injections after the total curfew was significantly reduced in comparison to the period during the total curfew (1% versus 3.5%; *P* = 0.063). The women were given a list of potential reasons for the inability to renew their contraceptive injections or IUD during and after the total curfew. During the total curfew, three women reported that the lockdown and clinics shutdown is responsible for the deficient continuation of their contraceptive injections or IUD; however, this number was reduced to 0 after the total curfew. None of the participants mentioned that financial reasons were responsible for the discontinuity of their IUD replacement during the total curfew, whereas only one woman reported that after the total curfew. The number of cases that were afraid to go to health centers for fear from getting infected with COVID-19 was reduced from six during the total curfew to two after the total curfew. Three of the women had deficient continuation of their injections or implants because of lack of childcare due to nurseries and school shutdown during the total curfew in comparison to one woman after the total curfew; two to three women reported that other reasons such as lack of transportation or absence of menstruation (because the time of menstruation is the preferred time for inserting IUD) contributed to the disruption of contraception injection or IUD repeat (Table 7).

**Table 7:** Proportion of participants who needed to replace or renew their implants or IUD

	During total curfew, % (*n*)	After total curfew, % (*n*)	*P*-value
Need to renew/change implant/injection/IUD	4.0 (8)	2.5 (5.0)	0.375
Did not manage to renew/change implant/injection/IUD	3.5 (7)	1.0 (2)	0.063
Reason for inability to renew/change implant/injection/IUD			
Lockdown and clinics shutdown	1.5 (3)	0.0 (0)	NA
Financial	0.0 (0)	0.5 (1)	NA
Fear from acquiring COVID-19	3.0 (6)	1.0 (2)	0.125
No childcare	1.5 (3)	0.5 (1)	0.5
Others	1.5 (3)	1.0 (2)	1.00

IUD = intra-uterine device; NA = not applicable.

## DISCUSSION

The global COVID-19 pandemic has nearly caused 1.7 million deaths, radically changed financial conditions, and disrupted many essential health services, including reproductive health services worldwide. This study grants the opportunity to evaluate the extent of the preparedness of the Jordanian healthcare systems in facing pandemic situations concerning reproductive health services.

Domestic offensive violence experienced by women is usually caused by various triggers such as stress, pandemics, emotional disruption, and drug and alcohol abuse. Abusive behaviors are variable, including physical, sexual, psychological, economic, or stalking behaviors.^11^ In the current study, 20.5% of the women suffered from increased domestic abuse during the COVID-19 pandemic. Similar previous studies at different parts of the world reported that domestic abuse and family violence have increased during the COVID-19 pandemic^12–16^ and in other similar epidemics such as Ebola and Zika viruses’ outbreaks.^17–19^ Peterman et al.^16^ stated that the increased incidence of domestic abuse during pandemics is explained by several factors such as poor financial safety, social isolation and quarantine, exploitative relationships, and reduced health and domestic support options. Most of these reasons are applicable to Jordanian community during the pandemic, especially during the early stages of the pandemic, which was associated with more time spent at home with partners, a complete loss of work or reduced income, and disruption of healthcare services that might have triggered the increase in domestic violence observed in this study.

Reports in the literature showing the impact of COVID-19 or even other pandemics or epidemics on birth canal health and menstruation are deficient, except for a very recent study which reported increased menstrual disorders but not vaginal infection during the COVID-19 pandemic.^20^ However, that study only involved a small number of participants (only 58 women) comparison to the current study, which involved 200 participants. The previous study results agree with the findings of the current study that after the total curfew in Jordan, COVID-19 was not associated with increased genital tract infection. Yuksel and Ozgor^20^ stated that the continual media concentration on personal hygiene to slow the spread of COVID‐19 was likely responsible for the prevention of reproductive tract infection. Similarly, these hygiene habits might also be responsible for the prevention of genital tract infection among women in Jordan, which is supported by a recent study indicating that the Jordanian community showed low-risk practices toward COVID-19, and the women in Jordan committed significantly better hygiene habits toward COVID-19 than men.^21^ By contrasts, other crisis, such as hurricanes and wars, were associated with increased genital tract infections due to limited access to health services, decreased hygiene, and loss of housing.^9,22–24^ Although limited access to health care was also present during the COVID-19 pandemic, decreased hygiene^21^ and loss of housing were not applicable in the Jordanian community during the pandemic, which might be responsible for the contradictory results. Moreover, contradicting results in the literature about the effect of stress on menstruation physiology and genital tract infection were reported. Some studies have shown that menstrual abnormalities were increased in some crisis, such as earthquakes and wars.^23–25^ Stressful events affect the female reproductive physiology at different levels, including the endocrine system, the autonomic nervous system, and the immune system, resulting in menstruation disorders.^26–29^ However, this is not the case in all stressful events because Singh et al.^30^ in their study showed that stressful events were not associated with menstrual irregularities. Surprisingly, this study found that the incidence of menstrual aberrations and genital tract infections among participants were significantly reduced during COVID-19–related total curfew, whereas the pre-pandemic state of menstrual disorders and gynecological infections was resumed after the total curfew. This variation in the results might be attributed to variations in population demographics, sample size, time of the study, or design of the study. Alternatively, the short curfew exposure time might be responsible for the contrasting results because it has been reported that the least exposure to stress was associated with less incidence of menstrual abnormalities,^25^ and the total curfew duration in Jordan was only for few continuous days. Furthermore, the high knowledge^31^ of the Jordanian population about COVID-19 during the early stages of the pandemic might have relieved their stress about this pandemic, resulting in decreased incidence of menstrual disorders and birth canal infections observed in the study. Moreover, measures taken by the government to contain COVID-19 transmission, mainly closing the borders, were associated with slow increase in the number of infected cases, with a total of 29 deaths and maximum of 77 cases/day being recorded during the study duration which was interrupted by zero cases for consecutive days in different occasions^32^ and might have also reduced the incidence of menstrual problems and genital tract infections. In addition, most of the infected cases were symptom free, discovered through random or exposed people sampling, and recovered completely. This might have also helped to relax the Jordanian community about the severity of the disease and may explain the reduced number of menstrual disorders and gynecological infections among women in Jordan.

In the present study, the proportion of women using contraception was significantly reduced during the total curfew. In addition, the proportion of women using contraception for family planning purposes was significantly reduced during the COVID-19 pandemic. This is similar to what was observed during previous viral outbreaks. For example, Ebola outbreak was associated with reduction in the utilization of family planning.^33,34^ A more recent study in Turkey has shown significant decline in using contraception during the COVID-19 pandemic.^20^ Similarly, during noninfectious crisis, the number of women using contraception was decreased significantly.^9,35^ This significant reduction in using contraception for family planning purpose is likely due to the complete lockdown of the maternity and childhood centers or health centers. These centers in Jordan provide contraception for nominal fees, and the proportion of the participants using these resources was declined significantly during the era of total curfew. Other possible reasons for reduced using contraception are the reduced supply due to disruption in the production or the shipping and distribution of the contraception products due to borders shutdown. As a consequence, the COVID-19 pandemic might be associated with increased incidence of unplanned pregnancy and adverse health outcomes in the future. This study demonstrates the need for better preparedness by the Jordanian healthcare system for better availability of better family planning services especially the emergency contraception ones during such critical situations because the consequence of disrupted family planning might be worse and continues longer than the effect of COVID-19 infection. This also requires an increase in the knowledge and awareness of Jordanian women about contraception methods that do not need external resources such as external ejaculation and fertility awareness, which can be applied when other contraception methods are deficient to reduce any unwanted adverse outcomes. Offering essential contraception techniques would definitely reduce devastating effects on women, their families, and the community.

In conclusion, the current work showed that the COVID-19 pandemic was associated with increased domestic abuse against women in Jordan. Moreover, pandemic-related curfew significantly reduced the incidence of menstrual disorders, genital tract infection, and the use of contraception, while the pre-pandemic state of menstruation, genital tract infection, and using contraception was resumed in the era of post-curfew. Moreover, contraception use and family planning during the pandemic were decreased as well. Therefore, these results could help in assessing the actual preparedness of the Jordanian healthcare system to introduce domestic support and reproductive health services and measures during pandemic situations. These results will constitute a general baseline to guide the relevant authorities in planning the required educational interventions and establishing effective caring techniques such as home visits or telemedicine for patients when the healthcare provider and patient are not physically meeting with each other.
